# Different Domains of Dengue Research in Malaysia: A Systematic Review and Meta-Analysis of Questionnaire-Based Studies

**DOI:** 10.3390/ijerph18094474

**Published:** 2021-04-23

**Authors:** Rhanye Mac Guad, Yuan Seng Wu, Yin Nwe Aung, Shamala Devi Sekaran, André Barretto Bruno Wilke, Wah Yun Low, Maw Shin Sim, Rogie Royce Carandang, Mohammad Saffree Jeffree, Hamed Taherdoost, Caroline Sunggip, Constance Liew Sat Lin, Chandrika Murugaiah, Vetriselvan Subramaniyan, Nornazirah Azizan

**Affiliations:** 1 Department of Pharmaceutical Life Sciences, Faculty of Pharmacy, Universiti Malaya, Kuala Lumpur 50603, Malaysia; garethsim@um.edu.my; 2 Department of Biomedical Science and Therapeutics, Faculty of Medicine and Health Science, Universiti Malaysia Sabah, Kota Kinabalu Sabah 88400, Malaysia; carolsun@ums.edu.my (C.S.); chandrika.murugaiah@ums.edu.my (C.M.); 3 Department of Biochemistry, School of Medicine, Faculty of Medicine, Bioscience and Nursing, MAHSA University, Selangor 42610, Malaysia; sengwu_21@yahoo.com; 4 Faculty of Medicine & Health Sciences, UCSI Hospital, 2, Avenue 3, Persiaran Springhill, Port Dickson, Negeri Sembilan, Negeri Sembilan 71010, Malaysia; yinna@ucsiuniversity.edu.my (Y.N.A.); shamala@ucsiuniversity.edu.my (S.D.S.); 5 Department of Public Health Sciences, Miller School of Medicine, University of Miami, Miami, FL 33136, USA; axb1737@med.miami.edu; 6 Faculty of Medicine, Universiti Malaya, Kuala Lumpur 50603, Malaysia; lowwy@um.edu.my; 7 Asia-Europe Institute, Universiti Malaya, Kuala Lumpur 50603, Malaysia; 8 Department of Community and Global Health, Graduate School of Medicine, The University of Tokyo, Tokyo 113-8654, Japan; rrcarandang@gmail.com; 9 Department of Community and Family Medicine, Faculty of Medicine and Health Science, Universiti Malaysia Sabah, Kota Kinabalu 88400, Malaysia; saffree@ums.edu.my; 10 Hamta Group, Research and Development Department, Research Club, Vancouver, BC 1211, Canada; hamed.taherdoost@gmail.com; 11 Hamta Group, Advanced Academic and Industrial Training Centre, Hamta Academy, Vancouver, BC 1211, Canada; 12 Medical Based Department, Faculty of Medicine & Health Science, Universiti Malaysia Sabah, Kota Kinabalu, Sabah 88400, Malaysia; constance.liew@ums.edu.my; 13 Department of Pharmacology, School of Medicine, Faculty of Medicine, Bioscience and Nursing, MAHSA University, Kuala Lumpur 42610, Malaysia; drvetriselvan@mahsa.edu.my; 14 Department of Pathobiology and Medical Diagnostics, Faculty of Medicine and Health Sciences, Universiti Malaysia Sabah, Kota Kinabalu 88400, Malaysia; nazirah@ums.edu.my

**Keywords:** dengue, Malaysia, survey, knowledge, attitude, practice, systematic review, meta-analysis

## Abstract

This review provided a systematic overview of the questionnaire-related dengue studies conducted in Malaysia and evaluated their reliability and validity used in the questionnaires. An extensive literature search was conducted using various electronic databases, including PubMed, EMBASE, Medline, and ScienceDirect. Systematic reviews and meta-analysis (PRISMA) were selected as the preferred item reporting method. Out of 88 identified dengue-related, 57 published from 2000 to April 2020 met the inclusion criteria and were included. Based on the meta-analysis, a poor mean score was obtained for knowledge (49%), attitude (44%), and preventive practice (55%). The study showed that the level of knowledge on cardinal signs and modes of transmission for dengue virus were highest among health care workers, followed by students (international and local) and lastly community residents. In treatment-seeking behaviours, only half of the respondents (50.8%) would send their child to the nearest health clinics or hospitals when a child became restless or lethargic. The acceptance rate for dengue vaccine, bacteria (Wolbachia), as a vector for dengue control and self-test diagnostic kit for dengue showed considerably high (88.4%, 70%, and 44.8%, respectively). Health belief model (HBM) constructs, such as perceived barriers, perceived severity, perceived susceptibility, self-efficacy, and perceived benefit influence prevention practices. Lastly, only 23 articles (40.3%) had piloted or pretested the questionnaire before surveying, in which three reported Cronbach’s alpha coefficient (0.70–0.90). A need for active participation of communities and healthcare personnel, promotion of awareness, and safe complementary medicines, as well as assessment of psychometric properties of questionnaire use in dengue surveys in Malaysia, in order for assessing dengue reliably and valid.

## 1. Introduction

Malaysia is situated in Southeast Asia and is comprised of thirteen states with three federal territories separated by the South China Sea into two regions, namely Peninsular Malaysia (also known as West Malaysia) and Borneo island (also known as East Malaysia) [1]. Over the past few decades, Malaysia has undergone tremendous technology-driven and high-tech production-based development in its economy and infrastructure, being one of the most developed countries in Asia [2]. However, the development is not equal in all states of Malaysia because some states are more urbanised than others. It has been reported that approximately two-thirds of the Malaysian population live in urban areas, making a substantial income gap between urban and rural communities. In fact, the Department of Statistic Malaysia (2011) reported that 3.8% of the 28.5 million people living in Malaysia live below the poverty line, mostly in states containing a relatively higher number of rural areas, such as Sabah, Sarawak, Perlis, and Kedah [3].

Alongside the rapid development and urbanisation, Malaysia has a high prevalence of diabetes mellitus [4], hypertension [5], and overweight/obesity (47.7%); thus, it is not surprising that these diseases contributed to 35% of all deaths in 2016 [6]. Urbanisation also leads to an increasing propagation of infectious diseases, including dengue fever, due to climatic change [7] and travel [8], as well as shifts in the range and distribution of dengue [8,9]. At present, dengue fever is considered a major public health concern in Malaysia, contributing to a high rate of morbidity and mortality [10]. However, the exact number of dengue cases in Malaysia is under-reported [11] partly due to the rapid population growth and the influx of a large number of foreign workers [12].

Malaysia is a multiethnic country with different cultures and traditions, consisting of Malay, Chinese, Indian, Orang Asli (mostly inhabiting in West Malaysia), and indigenous people of Sabah and Sarawak (e.g., Kadazan-dusun, Bajau, Iban, and Bidayuh). Over the years, the influx of foreign migrants has added to the diverse ethnicities, traditions and socioeconomic status of the country. Although the government imposed higher levies on skilled migrants [13], there have been recent amendments to this policy, whereby levies are also offered to skilled migrants for permanent residence or even citizenship. Apart from this, massive immigration was fastened to strengthen certain political parties [14]. This situation has created diverse populations merge of local and foreign cultures in Malaysia, further leading to complex inter-relationship in dengue transmission [15]. For example, statistics have reported that large numbers of unauthorised foreign migrants (locally simply known as “PATI”) in Sabah state, predominantly from the Philippines and Indonesia [16,17], could predispose a higher spread of dengue in this region.

Based on the abovementioned circumstances, the Vector-Borne Disease Control Programme under the Ministry of Health [18] has been implemented to formulate a dengue control programme, including surveillance and control, public education and inter-agency collaboration with community participation. Additionally, extensive research studies in various fields, such as epidemiological surveillance, laboratory diagnostic [19], clinical management, and entomological surveillance [20], have been conducted. In these research studies, various types of questionnaires have been designed to assess various domains, such as knowledge, attitude, and practice (KAP), treatment-seeking behaviour, relationship of health belief model (HBM) construct and health behaviour towards dengue, and perception towards dengue vaccine and complementary medicine (CAM) use for dengue.

Despite this, the collective scopes of these studies have never been discussed. The accuracy of findings from questionnaire-based studies is a matter of concern, as the extent of results’ accuracy largely depends on the reliability of the tools or questionnaires used in the study [21]. In this regard, a systematic review of questionnaire-related dengue studies is required to highlight the reported findings in this area, assess the validity and reliability of the questionnaires used in research, as well as draw broad conclusions. Thus, this systematic review aims to evaluate existing questionnaire-related dengue studies and its reliability and validity that may help to better characterise the questionnaire design and highlight future research needs in Malaysia.

## 2. Materials and Methods

### 2.1. Study Design

#### 2.1.1. Identification and Selection of Studies

**Figure 1:**
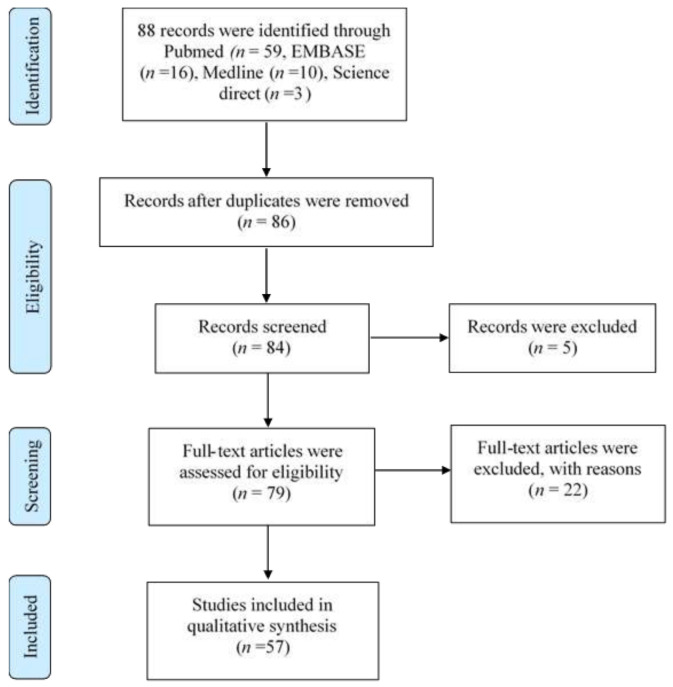
Flow diagram of identification, screening, eligibility, and included studies.

The methodology, research design, search strategy, and selection criteria were based on the review protocol developed by a panel researcher who comprise experts in clinical medicine, public health, and infectious diseases. An extensive literature search was conducted between March–June 2020 using various biomedical electronic databases, including PubMed, EMBASE, Medline, and ScienceDirect. Preferred reporting items for systematic reviews and meta-analysis (PRISMA) [22] were used for the exploration, as shown in Figure 1.

#### 2.1.2. Selection Criteria

Articles included in this review were peer-reviewed questionnaire-related dengue articles conducted in Malaysia and published in January–April 2020. All papers fulfilling the inclusion criteria were critically appraised by at least two reviewers independently based on the eight critical appraisals of CASP Checklist [23]. Any articles considered as part of the review were crosschecked through references and citations to ensure all relevant articles were included. Case report, conference proceedings and thesis were excluded due to lack of information for data extraction or peer-review evidence. The relevant articles were identified using keywords or a combination of strings encompassing ‘dengue’, ‘questionnaire’, and ‘Malaysia’. Additionally, a Boolean operator was used to link categories of the keyword, aiming to increase the query’s sensitivity and specificity.

#### 2.1.3. Data Extraction and Management

The decision to include any articles was made by reaching a consensus via email amongst the authors. A total of 88 articles were retrieved electronically, but after removing duplications, 11 articles were found either irrelevant or did not fulfil the abovementioned criteria and excluded. The remaining 77 articles were further assessed during the first round of review consisting of six expert reviewers based on the titles and abstracts, whereby five articles were eventually excluded. The second round of review was performed by three expert reviewers to ensure only relevant articles were included in the final selection based on the selection benchmark. Finally, a total of 57 articles were included in this review, and the flow chart of the research strategy is shown in Figure 1.

## 3. Results

### 3.1. Quantitative Analysis and Comparison of Recruited Questionnaire-Based Dengue Studies in Malaysia

**Figure 2:**
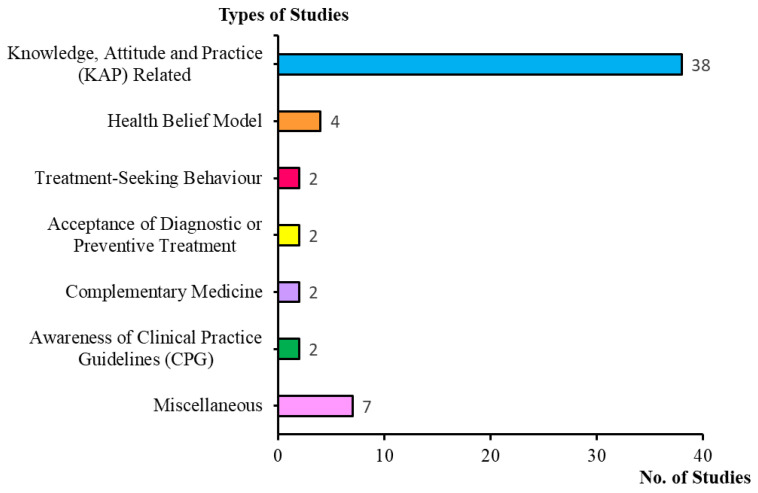
Type of questionnaire-related studies on dengue in Malaysia.

The exploration yielded 88 studies in all databases, but upon further review, only 57 studies were selected based on the selection criteria. Table 1 shows a summary of all extracted questionnaire-related dengue studies in which all of them are cross-sectional studies. Our results revealed that fewer research articles on dengue in Malaysia were published between the years 2003–2011. A significant increase in questionnaire-related dengue studies was conducted in Malaysia from 2013 onwards, constituting 92.9% of the total number of articles included in this review. Figure 2 depicts the various types of studies that were performed.

### 3.2. Location of Study

**Figure 3:**
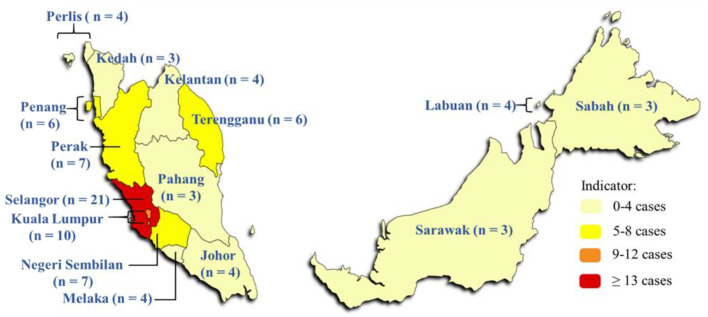
Number of questionnaire-based studies conducted in each state of Malaysia.

Almost all studies (94.7%) were conducted in West Malaysia, mainly in Selangor, Kuala Lumpur, Negeri Sembilan, and Perak, with three studies conducted nationwide (Figure 3). Parallel to dengue infection incidence, more than two-thirds were conducted in urban settings, with the target group of respondents from residential communities, health care workers and students. Only a few studies (12.3%) were conducted in a rural area amongst indigenous people or rural residential communities.

### 3.3. KAP towards Dengue Infection

**Figure 4:**
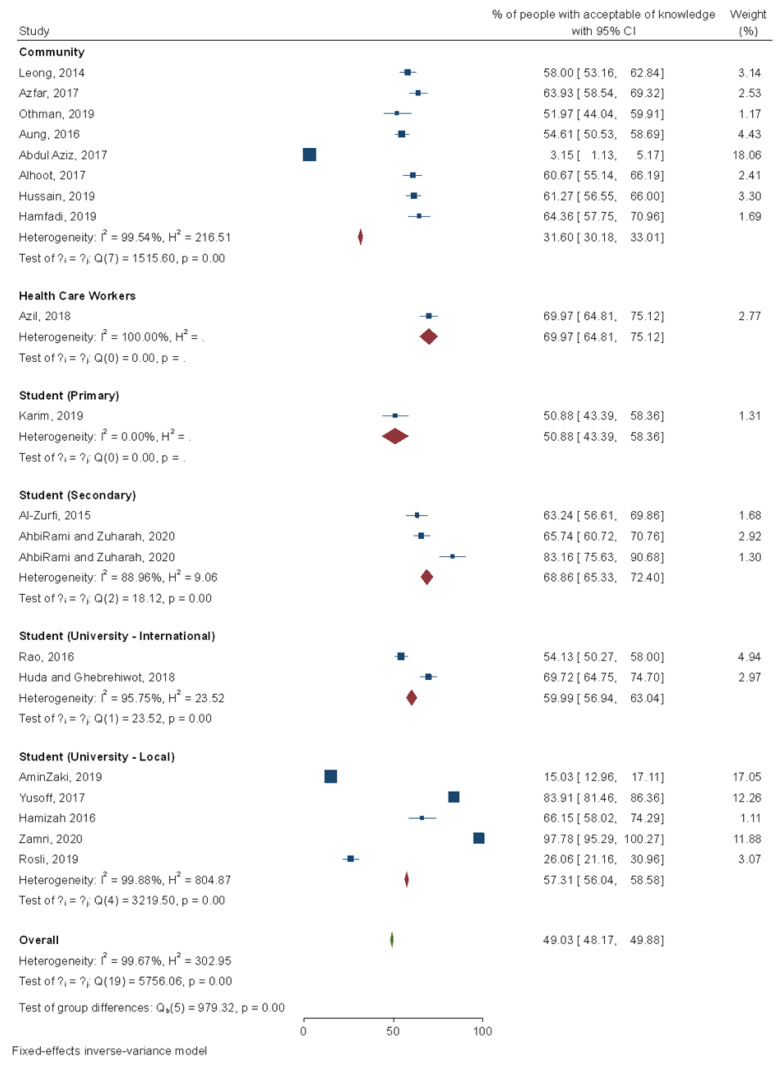
Meta-analysis of dengue knowledge scores in Malaysia. Note: I^2^ estimates the proportion of variation between the effect sizes due to heterogeneity relative to the pure sampling variation; I^2^ > 50 indicates substantial heterogeneity; H^2^ = 1 indicates perfect homogeneity among the studies; Q statistic is the weighted sum of squared differences between the observed effects and the weighted average effect.

A total of 20 studies reported on the percentage of the population with acceptable knowledge on dengue epidemiology (Figure 4). Upon meta-analysis, 49% (95% Confidence interval or CI: 48.2–49.9%) of Malaysia population have good knowledge of dengue. It was noticed that 31.6% (95% CI: 30.2–33.0%) of community people have an acceptable level of knowledge, and the proportion is increased to 70% (95% CI: 64.8–75.1%) if it comes to healthcare workers. Students have a higher proportion of good dengue knowledge as compared to community people. Among university students, international students exhibited a slightly higher proportion of having good knowledge but not significantly different. Amongst sociodemographic factors that have been reported to have a significant effect on dengue knowledge amongst respondents include age [46,49,57,59,71,74,76,77], gender [37,39,40,59], ethnicity [71,74], education level [24,37,57,59,71,76,77,79], marital status [46,49,77], living condition [64], type of house [64], occupation [35,48,49,64,77], monthly income [39,49,64], and personal history of dengue infection [40,79] or amongst family members [34]. Moreover, several studies have reported significant improvement in dengue knowledge amongst students [25,34,61,65] and healthcare personnel (*p* < 0.001) [73] after attending a health education program. An interesting finding by Burhanuddin et al. (2013) [76] was that the location of residents at different areas in Perak Tengah districts, Perak differed significantly in terms of their knowledge regarding dengue (*p* < 0.001). Amongst international postgraduate students studying in Malaysian universities, their length of stay in Malaysia was a significant predictor of their level of dengue knowledge (adjusted odd ratio (aOR) = 2.005, *p* = 0.007) [40].

**Figure 5:**
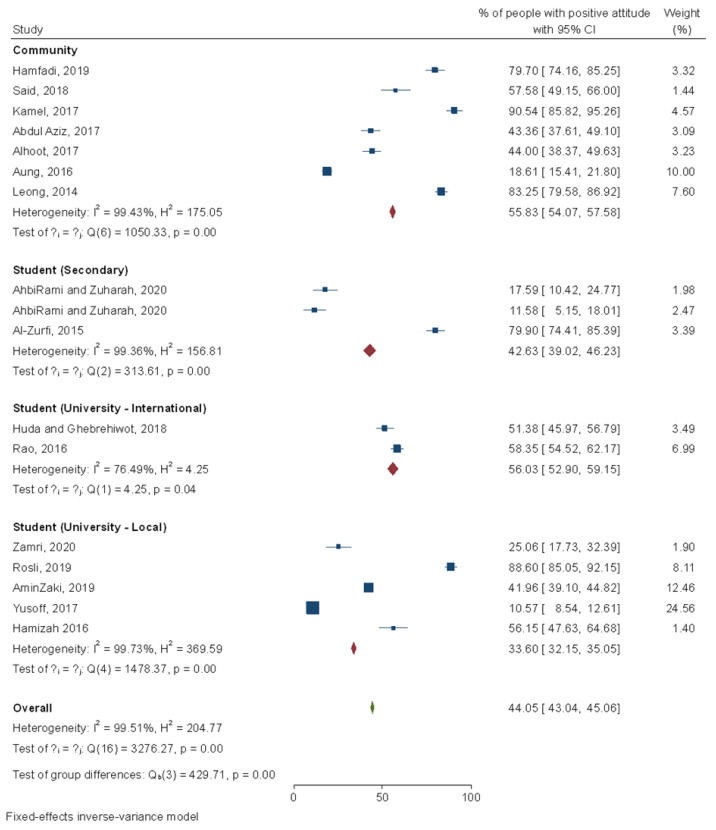
Meta-analysis of attitude scores among Malaysian communities. Note: I^2^ estimates the proportion of variation between the effect sizes due to heterogeneity relative to the pure sampling variation; I^2^ > 50 indicates substantial heterogeneity; H^2^ = 1 indicates perfect homogeneity among the studies; Q statistic is the weighted sum of squared differences between the observed effects and the weighted average effect.

Next, 17 studies have reported the attitude of respondents towards dengue. It was found that 44.1% of the population had a good attitude towards dengue where more than half of the community (55.8%, 95% CI: 54.1–57.6%) and university international students (56.0%, 95% CI: 52.9–59.2%) had a good attitude, while only one-third of the local university students (33.6%, 95% CI: 32.2–35.1%) showed good attitude. Further analysis discovered that attitude towards dengue was associated with age [40], ethnicity [41,71], gender [79], education level [24,51,67,68,71,74], knowledge on dengue [24,36,41,45,48,55,62,68,71,72,74], health campaign [40], occupation [48,74], and monthly income [51]. Amongst international postgraduate students in a Malaysian university, their attitude was significantly associated with the country of origin [40] (Figure 5).

**Figure 6:**
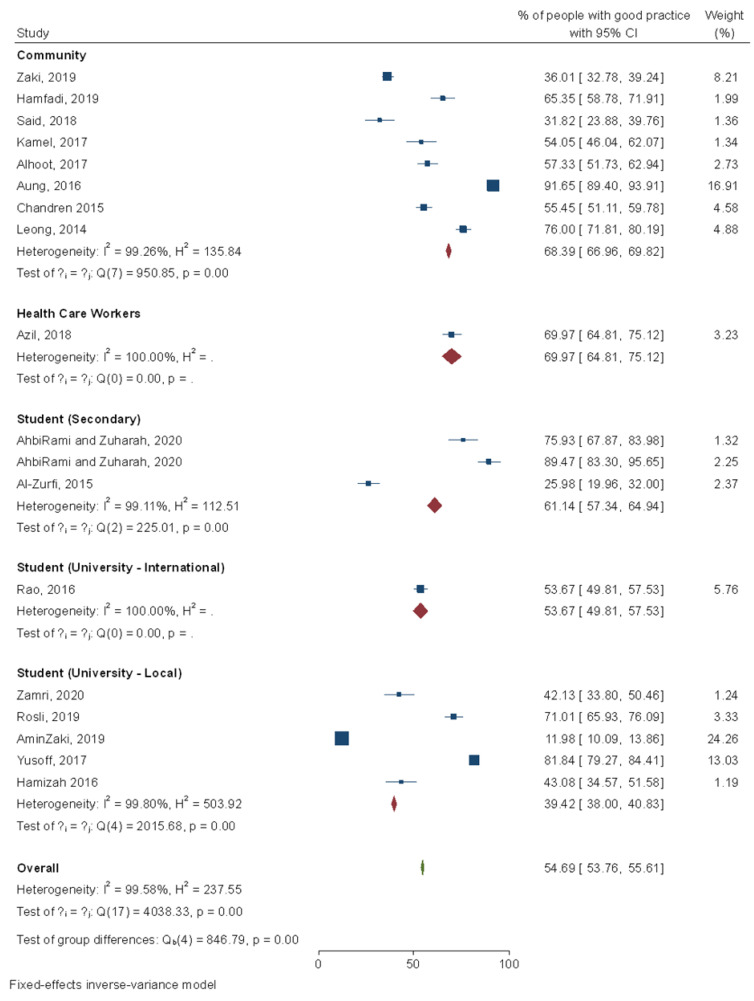
Meta-analysis of dengue practice score in Malaysia. Note: I^2^ estimates the proportion of variation between the effect sizes due to heterogeneity relative to the pure sampling variation; I^2^ > 50 indicates substantial heterogeneity; H^2^ = 1 indicates perfect homogeneity among the studies; Q statistic is the weighted sum of squared differences between the observed effects and the weighted average effect.

From 18 studies reporting on practice towards dengue prevention, 55% (54.7%, 95% CI: 53.8–55.6%) of the population had a good practice on dengue prevention, regardless of the poor percentage (<50%) of people with acceptable knowledge on dengue. Meanwhile, an interesting finding was noticed among local university students. Although a higher proportion of them with good knowledge, most (60.8%, 95% CI: 59.2–62%) of them did not adopt good practice. On the other hand, age [25,46,47,52,56,57,59,63,74,77], gender [47,55,56,59], occupation [46,48,63], marital status [40,46,47,55,56,71,74], location of residential house [47,74,79], levels of education [35,38,39,47,56,59,77], history of infection [25,35], monthly income [39,52], attendance to health campaign [55], knowledge on dengue [24,27,31,35,38,43,46,47,48,55,57,62,64,71,74,77,79,80], ethnicity [59,71], experience of fogging [64], and house ownership [79] have been significantly associated with dengue preventive practices. Additionally, the duration of stay at the university hostel amongst international students showed a significant association with their preventive dengue practices [40] (*p* = 0.049) (Figure 6).

### 3.4. Treatment-Seeking Behaviour

A study carried out by Fatini et al. [52] in dengue-endemic areas of Kubang Kerian Kelantan reported that most respondents (70.6%) would visit the nearest hospital immediately. However, half of the respondents would take antipyretic themselves or wait for a few hours whether symptoms had improved (30%) before visiting a hospital. Similarly, Ariffin et al. [69] reported common treatment-seeking behaviours amongst parents or caretakers, including giving antipyretics (3.6%) or waiting for improvement before sending an infected dengue individual to a hospital (7.8%). Interestingly, no parents or caretakers would call an ambulance. Worryingly, despite this life-threatening condition, only half of the respondents (50.8%) would send their child to the nearest health clinics or hospitals when a child became restless or lethargic. Besides, some common factors associated with treatment-seeking behaviour were monthly income or history of dengue hospitalisation (*p* = 0.04).

### 3.5. Acceptance of Diagnostic and Preventive Treatment

Yeo et al. [41] reported that the acceptance of a dengue vaccine was high (88.4%) among a population in Penang, Malaysia. The study further concluded that the acceptance of the dengue vaccine was significantly associated with Chinese ethnicity (OR = 0.36, *p* = 0.017), levels of dengue knowledge (OR = 1.43, *p* = 0.016), and vaccination attitude (OR = 1.91, *p* < 0.001).

Next, a study conducted amongst 330 healthcare providers from the University Kebangsaan Malaysia Medical Centre (UKMMC) showed a high level of acceptance towards bacteria (*Wolbachia*) as a vector for dengue control (70%). The acceptance of using this type of dengue control was significantly associated with occupation (OR = 8.83; 95% CI = 2.60–29.96) and levels of dengue knowledge [39] (OR = 6.07, 95% CI = 2.89–12.74).

Besides, a nationwide study through telephone survey was conducted by Wong et al. [68] involving 2512 Malaysian aged 18–60 years old showed a favourable acceptance of a self-test diagnostic kit for dengue at home (44.8%). Interestingly, further analysis revealed that the acceptance was significantly associated with the levels of education (OR = 0.65, 95% CI = 0.43–0.89, *p* = 0.02), perceived barriers to dengue prevention (OR = 0.67, 95% CI = 0.53–0.85, *p* < 0.001), and dengue knowledge (OR = 0.75, 95% CI = 0.61–0.91, *p* = 0.001).

### 3.6. CAM for Dengue Prevention

Most dengue patients (85.3%) showed a favourable acceptance towards the use of CAM as reported by Ching et al. (2015) [67]. It was further noted that the most popular CAM used was isotonic drinks (85.8%), crab soup (46.7%), and papaya leaf extract (22.2%). They also discovered that the reasons contributed to the use of CAM was due to a good impression on CAM from other CAM users (33.3%, mostly by their family members) or some just wanted to try it (32.2%), while having completed tertiary education was more likely to use CAM as compared to secondary level and primary and below (OR = 5.8, 95% CI = 1.62–20.45, *p* = 0.01).

### 3.7. Health Belief Association with Dengue Infection

Several studies have indicated a significant association of dengue infection with health belief models. For example, in a study involving 560 Orang Asli indigenous people living in a rural area in Peninsular Malaysia, discovered that respondents with low perceived barriers towards dengue (OR = 2.06, 95%CI = 1.21–3.53, *p* = 0.008) were more likely to practise better prevention practices [64].

A study performed by Othman, et al. (2019) [27] reported that most respondents (65.1%) felt distressed when a member of the household is having a continuous fever (perceived severity), wherein 57.9% of them believed that they are all vulnerable to dengue, 77.6% worried if they found any standing water surrounding their premises (perceived susceptibility), and 57.9% admitted having sufficient knowledge on dengue and Aedes mosquito. Furthermore, 60.5% agreed that they will ensure no breeding sites in their premises, while 42.1% of respondents had played their roles in controlling dengue in their areas (self-efficacy). The study also discovered that 2.6% perceived themselves as having other important tasks that caused them unable to convey information about dengue to their family (perceived barrier), while 58.6% agreed that they will benefit after practising all preventive measures. Lastly, 53.3% believed that disseminating the knowledge of dengue and Aedes mosquito could help to save somebody from dengue infection (perceived benefit).

In another study conducted by Abd Rahman et al. (2020) [70], they reported that despite having good knowledge (70–90%), 47% of the respondents agreed that Aedes mosquitoes do not bite in the day time either they do not feel the bite (53%), bitten regularly by mosquitoes (61.1%), or having a healthy body (56.5%) (perceived susceptibility). Furthermore, more than two-thirds of respondents (74.9%) agreed that dengue is a serious condition (perceived severity), with >80% of them perceived that fogging activities and involvement in health campaigns could help to reduce dengue (perceived benefits). In comparison, one-third of the respondents (39.5%) considered that thermal and ultra-low volume (ULV) fogging are harmful to health (perceived barrier).

Similarly, a study carried out by Kai et al. (2019) [33] involving 322 heterogeneous urban populations in Kuala Lumpur showed that, despite having good knowledge, 48.6% did not check the current dengue situations or hotspots around their area regularly (perceived susceptibility), while 46% felt that chemical fogging by the local authority is good enough to prevent dengue infection (perceived benefits). Interestingly, 73.6% of the total respondents who would take part in a public activity for dengue control (perceived benefits), among which 64% would search and destroy mosquito breeding sites or removing mosquito breeding sites (perceived barrier).

Apart from these, a nationwide study amongst resident communities within 3 km radius from schools in Malaysia [60] reported a significant association between dengue seroprevalence using Immunoglobulin G (IgG) antibodies seropositivity and their perceived severity (adjusted OR = 1.84, (95% CI = 1.25–2.87), perceived susceptibility (adjusted OR = 4.50, 95% CI = 1.95–10.99), or perceived barriers (adjusted OR = 0.85, 95% CI = 0.35–1.29) to dengue prevention.

### 3.8. Awareness and Utilization of Clinical Practice Guidelines (CPG)

Amongst health practitioners, it was shown that the level of awareness on Clinical Practice Guidelines (CPG) was higher amongst public health practitioners (99%) as compared to private practitioners (84%) [53]. Not surprisingly, the percentage of Medical Officer in private facilities utilising CPG was lower than those from public facilities (84% versus 98%, respectively).

### 3.9. Sources of Information on Dengue

Regarding the source of receiving dengue information, a study by Ng et al. (2016) [61] involving secondary school students showed that television (93.7%), newspaper (90.5%), and the internet (78%) were the commonest sources of attaining dengue information. Comparatively, social media (e.g., Facebook, Twitter, Instagram, and Snapchat) (44%) [29], together with television and radio [37,57,74], are the popular medium to disseminate dengue information amongst university students.

On the other hand, amongst community residents of urban, semi-urban and rural areas, they mostly obtained dengue information from television (97.0%), printed media (74.3%), and radio (63.7%), while the least reported source of information was through talks and seminars (29.7%) [74]. When stratified into the locality of the residents, Othman et al. (2019) [27] revealed that the commonest informants for community residents in an urban dengue epidemic area in Selangor were television or radio (89.8%), which is higher than the findings (radio, 71.4%; television, 41.3%) of Kai et al. (2019) [33] amongst urban communities in Kuala Lumpur.

Interestingly, it was also found that the preferred sources of dengue information among rural communities were similar to urban communities; (i) television and radio (82.0%), followed by information from their relatives/friends (57.5%) or pamphlets/posters (34.5%) in Kuala Kangsar Perak [80]; (ii) mass media (90.1%), health campaign (90.6%), newspaper (81.6%), and neighbours (73.4%) in Terengganu [57]; and (iii) television or radio (88.5%) in Rembau Negeri Sembilan [71].

Lastly, a recent study implemented by Mahalingam et al. (2019) [30] showed that the use of modern technology, such as e-learning through smartphones (97.6%) to access dengue information, amongst community residents was influenced by several elements, including ethnicity, age, education level, total household income, or the number of dependents in a family. Similarly, the use of the internet (18.6%) in addition to television or radio (32.8%) was the most widespread source of dengue information amongst the military cadets [66].

### 3.10. Reliability and Validity of the Questionnaire

Out of 57 articles reviewed, 22 articles have piloted or pretested the questionnaire [24,29,34,36,38,41,48,50,53,57,63,65,66,67,68,71,72,73,75,77,78,79] before surveying. A total of 14 articles [26,30,31,33,35,37,42,43,47,51,54,62,74,80] were considered as having a high risk of bias on the questionnaire due to lack of evidence on validity and reliability (data not shown), among which two articles were adapted directly from the similar previous study, and 10 articles were a newly developed questionnaire.

## 4. Discussion

There are no specific treatments for dengue, and the current vaccine has limited applications due to its moderate efficacy, not providing equal protection against all dengue serotypes [81]. Hence, the only mainstay of dengue treatment is through vector control. In this regard, human practice is known to play a pivotal role in maintaining vector control and transmission. To the best of our knowledge, this systematic review is the first to provide an overview of various aspects in all questionnaire-related dengue studies conducted in Malaysia between 2000–2020 where an upsurge of dengue cases has been reported over the years. Additionally, this review highlighted the information on the lack of evidence on the reliability and validity of the questionnaire used in the studies.

### 4.1. Knowledge, Attitude, and Practice towards Dengue Infection

Overall, the analysis in this review showed that the respondents had sufficient KAP regarding dengue in Malaysia. The level of KAP is comparable to other studies conducted in Southeast Asia countries, including the Philippines [82], Indonesia [83], Thailand [84], and India [85]. One of the reasons for the comparable findings could be due to the occurrence and mortality of dengue in these tropical countries that affect their knowledge level [83]. As a consequence, a tremendous effort by the health department in Malaysia is essential for controlling dengue, especially in areas where the disease has been the most severe. One of the efforts that could be implemented is health education campaigns might lead to an increase in knowledge regarding dengue as reported in Myanmar [86] and Indonesia [87].

Nonetheless, it should also be noted that this review demonstrated that the Malaysian population had adequate knowledge of dengue infection per se on signs and symptoms. Some common problems in lacking dengue knowledge are misconceptions of dengue transmission (e.g., breed in dirty water, preferred biting time, and identification of typical symptoms of dengue) [48,77], treatment or prevention. Presumably, the respondents could not state typical symptoms of dengue because they neither personally experienced the disease nor witnessed a case from a close relative or member of the community or due to lacking regular awareness programmes on dengue infection in their area. This presumption is consistent with earlier cross-sectional studies in Indonesia [88] and India [89] that could lead to failure in controlling the infection and a delay in medical intervention. Moreover, this situation might be worsening by lacking the knowledge of the vector’s habit, behaviour and life cycle as reported in this review. In fact, other similar studies have also expressed the same, such as Thailand [90]. Many studies have reported several factors associated with dengue knowledge, suggesting a role of both health authorities and community residents in spearheading dengue prevention program.

In this review, most respondents demonstrated a good attitude, reflecting that they had perceived risk of dengue and supported dengue control. In addition to the similar associated factors affecting attitude towards dengue as reported in other countries, this finding might also be partially influenced by the local Malaysian culture of trying to please any guests because the surveys were conducted by self-administered (63.2%), face-to-face interviewer (33.3%), and phone call interview (3.5%) using a structured questionnaire, indicating possible cultural influence as reported in a study in Nepal [91].

In Sabah, the rising incidence of severe dengue has been reported in two main eastern districts, namely Tawau and Sandakan [92]. Interestingly, these regions are mostly associated with illegal migrants from the Philippines and Indonesia [93]. It could be partly due to sociocultural factors, including human movement patterns, whereby these populations lived in a poor condition that is suitable for dengue infection. However, no study has been conducted to compare KAP between locals and migrants in Malaysia; thus, it would be interesting to assess these populations, as reports have associated migrants with the transmission of infectious diseases [94].

Another interesting finding in this review is that despite a significant association between knowledge of dengue and attitude towards Aedes mosquito control, some studies have reported that good knowledge and good practice may be unparallel. However, other studies have reported similar levels of association [95,96], with few others citing positive association [97,98]. Although the exact factor that inhibits the translation of dengue knowledge to preventive practice is unknown, we hypothesised several major factors based on the rationale and analysis of the reviewed articles. Firstly, this situation could be due to the lack of regular and continuous awareness programmes conducted in the area that leads to discrepancies in their attitude or preventive practices. Secondly, an effective practice towards dengue prevention might be affected by traditional beliefs and culture of a certain ethnicity. For instance, it has been shown that rural Malay ethnic living in a traditional lifestyle spent large amounts of time in and around the house, resulting in positive attitudes towards the elimination of mosquito breeding sites [48]. Furthermore, people may resist household or personal practices to control the vector and considering that it is the responsibility of the government. A recent study indicated that the impact of uncontrolled and intensified deforestation on increased dengue cases in Sabah due to sylvatic spillage of dengue virus [92], suggesting further investigation could focus on specific demographic factors and land-use contributing to dengue breeding.

The review provides several important steps in dengue prevention and control. Firstly, future dengue awareness campaigns should be targeted at communities in both endemic and non-endemic areas throughout Malaysia. Secondly, the process of transformation related to their traditional beliefs on health requires continuous processes and integrated cooperation between government (e.g., Health Departments and Department of Occupational Safety and Health of Malaysia), non-government organisation, district office, head of the village (ketua kampung in Malay language), and Village Development and Security Committee (Jawatankuasa Kemajuan dan Keselamatan Kampung in Malay language). Thirdly, active participation and effective communication should be fostered when conducting regular voluntary communal works, aiming to motivate and strengthen a healthy lifestyle, such as the integration of appropriate dengue prevention practices. Besides, in this sort of setting, conventional education campaign to increase dengue awareness is useful, but the programme materials need to be evaluated on a routine basis, including health messages content, as well as training of personnel to deliver educational messages. In fact, a study in Cambodia [99] revealed that the content of the materials in health pamphlets was not always practical or effective in preventing mosquito bites, leading to difficulty in knowledge translation to the communities. In addition, when designing health pamphlets regarding dengue, the level of literacy and cognitive understanding of the concerned population should be considered and incorporated in the final product [100]. Lastly, ingrained and negative habits are difficult to discourage with plain knowledge sharing. Perhaps more personal and practical approaches in the health education programmes are required to stimulate behavioural change, including co-operation with faith-based organisations, particularly in rural areas, in Malaysia.

### 4.2. Treatment-Seeking Behaviour

Despite the importance of immediate treatment-seeking behaviour upon dengue infection, only a few studies have been conducted in Malaysia. Similar patterns of treatment-seeking behaviour have been demonstrated in Myanmar [101] and Venezuela [102], reflecting a lack of awareness of the need for immediate medical attention for dengue. This situation could be caused by several factors, including: (i) misconception that dengue is easily curable without progression to further complications, and this intention is worrying; (ii) poor transportation amongst rural populations in certain states of Malaysia, such as Sabah and Sarawak; (iii) poor healthcare services, such as poor ambulance response time in case of emergency, lack of facilities, and low skills of health care providers, that lead to lack of confidence among community residents [103,104,105] could partly explain the reason for a delay in treatment-seeking behaviour among Malaysian population; and (iv) dependency to symptom-relief-based supporting therapy, especially over-the-counter medication like paracetamol [106].

The findings in this review suggested several recommendations to improve the dengue treatment-seeking behaviour. Firstly, comprehensive information on dengue should be included in health education at all levels in the community, including school-aged children [61], regarding the critical of seeking immediate treatment through raising awareness on the importance of early attendance to health centres. This recommendation is vital to populations at higher risk of dengue infection, such as “sociodemographic-deprived” and history of dengue hospitalisation, due to the fact that they are prone to a higher risk of clinical complications, such as severe dengue, and even death. Besides, home-based treatment, such as the use of paracetamol or traditional medicines, needs to be assessed for efficacy and safety as it is widely perceived and experienced as efficacious for treating dengue in Malaysia, especially among rural populations [107]. This is because a multi-centre randomised controlled trial demonstrated that the use of standard-dose paracetamol in dengue infection increased the incidence of transaminase elevation in the liver and clinical complications, such as gastric haemorrhage and acute kidney injury [108].

### 4.3. Acceptance of Diagnostic and Preventive Treatment

Based on a study by Yeo et al. (2018) (40), the rate of dengue vaccine acceptance in a subset of the Malaysia population was lower than a similar study in Bandung, Indonesia (88.4% vs. 95%) [109]. This information on dengue acceptance is important as one of the criteria that World Health Organisation (WHO) recommends for vaccine implementation in a population [110]. However, further study is warranted to assess the strength of the country’s current immunisation system and the characteristics of the vaccine itself.

Overall, this review showed that the level of acceptance towards diagnostic or preventive treatment, such as dengue vaccine, bacteria (Wolbachia) as a vector for dengue control, and self-test diagnostic kit for dengue, was significantly associated with the level of dengue knowledge. Theoretically, people with higher dengue knowledge are more aware of any information, including the benefits of vaccination, limitations of treatment, and the importance of early dengue detection, such as using a home self-use kit. Similar association has also been demonstrated in other infectious diseases, such as the human papillomavirus vaccine [111] and influenza [112].

Additionally, the acceptance of a hypothetical dengue vaccine was significantly associated with their attitude towards vaccination practice in Malaysia, suggesting a need to improve their attitude as it has a more discernible effect on increasing vaccine acceptance. Similar findings have been reported in the Indonesian population [109]. Furthermore, a study in Indonesia [113] demonstrated the importance of parental acceptance on the school children’s vaccination programme as reflected in national coverage of the Expanded Programme of Immunisation (EPI) vaccination (>90% amongst infants and school children). It has been reported that other populations also adopted a similar attitude towards vaccination against other infectious diseases, such as human papillomavirus [114], influenza A virus subtype H1N1 [115], rubella, and measles [116]. Furthermore, the acceptability of the dengue vaccine is also associated with trust in public health institutions, vaccination programmes, and health institutions, making them accept the vaccine rooted in the controversial vaccine experience as shown by the Philippines population [117].

Given the aforementioned situation, it is therefore deemed essential to increase public health trust by any means via incorporation of community participation, health promotion programme, and informal social networks in disseminating public health messages. Interesting findings in a unique Malaysia multi-ethnicity country showed the importance of ethnicity in the acceptance of a hypothetical dengue vaccine [41]. It is possible that the differences are not due to ethnicity per se but due to other sociodemographic characteristics, such as age, gender, level of education, or occupation. The disparities across the three main ethnic groups in the use of dengue home test kits if available would be interesting to investigate further.

### 4.4. CAM as Dengue Treatment

It was reported that a high number of dengue patients in the central region of Peninsular Malaysia depend on complementary medicine (85.3%), which is similar to the East Coast of Peninsular Malaysia (84.6%) [107]. Interestingly, the reported prevalence rate was found lower in an earlier study involving all states in Malaysia (69.4%) [118], Japan (76%) [119], South Korea (ranged 29% to 83%) [120], and Singapore (76%) [121]. The variations in the prevalence may be due to one or multiple factors, including methodological criteria of assessment (e.g., age of the population examined, locality of the studied region and CAM definition) and the purpose of using CAM for health maintenance rather than for treating illness.

According to the review findings, the use of CAM, such as papaya leaf extract (Carica papaya), was increased amongst dengue patients, particularly among rural populations due to economic and geographical constraints [122]. Similar findings have been shown in other populations in Indonesia [123], the Philippines [124], and India [125]. Clinical studies have reported the beneficial effects of C. papaya on dengue patients by improving their platelet counts, reducing inflammatory reactions [126] and maintaining the stability of the haematocrit level [123]. Arguably, this finding did not correlate with life-threatening complications of dengue infection, such as plasma leakage and shock syndrome [127]. Therefore, the use of C. papaya is limited as acute and temporary dengue management for rural populations while waiting for transportation to receive hospital management. Similarly, the use of isotonic drink did not reduce dengue severity other than giving supportive effects helping dengue patients to regain energy. In view of diabetes, it may endanger the life of a diabetic patient infected with dengue due to its high sugar content [128] if over-consumed.

With the advancement of molecular techniques, a more comprehensive study on the association of different genes in determining other roles of C. papaya is warranted in order to justify the value of conducting a dengue awareness campaign on CAM in Malaysia. Alternatively, leaves extract of C. papaya has been demonstrated to have larvicidal activity against mosquito Aedes, the primary dengue vector for dengue infection, suggesting an ideal approach for vector control programme [129]. Although reports have concluded that the administration of C. papaya is safe [130], further study is required to elucidate its mechanistic action in providing alternative or adjuvant therapy to dengue patients.

Lastly, the analysis in this review also discovered that an association between the use of CAM and higher level of education, which is consistent with another local study among dengue patients in the northeast region of Peninsular Malaysia [107]. This finding may be due to the fact that they are more aware of dengue complications, making them search for health supplements to complement the treatment received in the hospital. Similar findings were also reported in other populations [120].

### 4.5. HBM Construct Association with Dengue Prevention Practices

HBM constructs can be utilised to predict people’s behaviour or why people take action to control or prevent a specific illness or disease. They can guide the plan of dengue interventions and the development of an effective awareness or targeted educational programme in Malaysia. However, only six out of 57 studies in this review considered HBM constructs and not all of them correlate to attitude and practices.

Multiple studies have indicated that the practice of protective behaviours is more dependent on the beliefs based on HBM constructs. Although only one study correlated the HBM constructs with prevention practices, the perceived barriers and perceived susceptibility constructs of the HBM were significantly related to prevention practices [62]. People with lower perceived barriers were significantly related to higher dengue prevention practices [62]. To perform dengue prevention effectively, it is imperative to eliminate barriers hindering actions against dengue. For instance, a qualitative study conducted amongst the Indonesian population [131] demonstrated the importance of clarifying appropriate action and promoting positive effects on performing dengue vector control to reduce persistent dengue transmission. Similar to cancers, another study among female students in Botswana, Southern Africa, showed that misconception could act as a barrier to breast cancer screening [132], indicating the importance of identifying and reducing perceived barriers through interventional programmes to correct health misinformation.

In addition, people with lower perceived susceptibility to dengue were less possible to carry out dengue prevention practices, most probably due to Orang Asli are either not aware of the serious consequences of dengue fever or never experienced themselves. Previous studies have revealed that if action is expected to occur, the individual perceives the susceptibility of having the illness [133]. A recent study in the dengue-endemic city Karachi, Pakistan [134] showed that perceived threat was significantly associated (*p* = 0.000–0.007) with acquiring information on dengue knowledge among populations as compared to low intensity to acquire knowledge on dengue in a non-endemic area of dengue in Malaysia [33]. This finding is important because it is a prerequisite for population stratification based on risk levels, personalise risk based on a person’s characteristics or behaviour, making perceived susceptibility more consistent with individuals’ actual risk or specify consequences of risks and conditions.

In this review, only one study related HBM concepts with attitude, and they found that the highest satisfactory attitude was in the perceived benefit concept [26]. Hence, this study highlighted that health education, campaigns and knowledge of dengue fever were highly required and essential in order to improve preventive practices among communities, particularly Orang Asli. It was further suggested that health messages and education awareness campaigns should be prepared in accordance with HBM constructs.

Based on the above, it is noted that HBM plays an important role and should be used as the predominant theory-driven intervention strategies to reduce dengue. However, in interpreting the relationship of HBM constructs as a conceptual guiding framework for health behaviour intervention, caution must be exercised when drawing a conclusion as HBM has some limitations. One example is that HBM does not account for environmental factors that may prevent an individual from practising the desired preventive practices, including poor infrastructure, bad sanitation, and inadequate water supply experienced by community residents. Thus, it is essential for the government and private sectors to work co-operatively providing facilities to remove the barriers in conjunction with behavioural change interventions of the community for sustainable dengue prevention and control.

### 4.6. Awareness and Utilization of Clinical Practice Guidelines (CPG)

The Ministry of Health in Malaysia has adapted the CPG prepared by the WHO for managing dengue, with evidence of good awareness by clinicians. Despite quantitative survey evidence on a high level of dengue awareness regarding CPG amongst medical doctors in Malaysia [53], the study showed differences amongst medical practitioners in public and private health facilities. It could be explained that medical doctors in public health facilities are younger, thus they are more likely to look for guidance in managing their patients. Comparatively, those working as private health practitioners tend to undermine their well-experienced clinical practice in managing their patients.

Nonetheless, several studies in other countries [135,136,137] have demonstrated that the recommendations stated in the CPG are impractical in some clinical settings, including limitations in consulting time, lack of resources, lack of attention to the logistics of implementation, or lack of detail on particular patient groups. Due to this, the adaptation of ‘local’ guidelines in public health facilities with highly dengue admission rate has been reported, which generally advocated fixed regimes of fluid management with limited resources [138].

Although public facilities continuously conduct training to their staff regarding CPG to enhance their awareness and implementation, the use of standard CPG could result in a problematic situation in a patient requiring more tailored fluid regimes. Therefore, the final decision on the implementation of guidelines in routine patient management should be evaluated thoroughly to maximise the benefits to the patients. Importantly, providing guidelines in clinical settings should be in line with the emerging model of translational science in healthcare, which integrates translational research and effectiveness a novel and timely evidence-based revision [139] for maximal benefit of the patients.

### 4.7. Sources of Information on Dengue

In this review, the analysis showed that most Malaysians obtained information regarding dengue from mass media, such as television, radio, and newspaper, suggesting that this platform is important in disseminating dengue-related information to raise better awareness. Additionally, more audiences are increasingly engaging with online social media, including Facebook, Instagram, Twitter and other smartphone dating applications. The obtained dengue-related information could be utilised for health promotion and would be a promising method due to relatively inexpensive, convenient accessibility and a fast approach. However, astoundingly, over 72% of Malaysian national ground territory seemingly has near-zero telecommunication coverage, with a major void even in the central Malaysian peninsula [140], that could create information barriers. Moreover, one should be aware of the fact that not all the information accessible through social media is credible.

Recent studies have suggested that dengue outbreaks are increasing in rural settings of Sabah due to urbanisation and deforestation [92], thus increasing dengue awareness and preventive practices are urgently needed in this area. An effective method of delivering dengue information in rural areas would be a community-based health education delivered by nearby health center outreach activities and health campaigns as demonstrated amongst rural population in Cambodia [99]. However, the outcome from the previous study recommended that a more persuasive approach, such as modelling of performing the desired behaviours or relating their personal sufferings due to their attitude and practices, should be incorporated in health education as it could form better confidence amongst respondents [141]. Additionally, health education could be conducted by healthcare providers to provide dengue-related speeches in formal or non-formal religious lecture sessions in religious centres, such as mosques, churches, or temples, as it has been shown to provide a similar opportunity for increasing dengue awareness in Indonesia [83].

Surprisingly, this review showed that healthcare providers demonstrated a lower function as a source of attaining dengue-related information amongst respondents. This issue merits a further examination, as they are considered as the frontliners that highlight the importance of prompt treatment of dengue patients. This finding is opposed to a study in Aceh, Indonesia, that healthcare providers had been cited as a major source of information on dengue [83]. The contradicting finding could be due to patients perceived trustworthiness and acceptance of healthcare services as reported in Uganda, Africa [142]. Plausibly, the Communications for Behavioural Changes (COMBI) programme on dengue prevention implemented by Health Departments and developers at construction sites to promote dengue awareness and prevention failed to achieve desired behavioural impact among community [143], which may be due to lacking respectful and supportive patient-provider interactions.

### 4.8. Lack of Evidence in the Questionnaire’s Reliability and Validity

Validity and reliability are very important to measure the accuracy and consistency of research tools, particularly questionnaires. However, their measure is not regularly conducted among healthcare researchers in developing countries. The reliability of a questionnaire is usually measured using a pilot test. Moreover, the reliability could be assessed in three major forms, including test-retest reliability, alternate-form reliability and internal consistency reliability. It is important to take note that some studies have mentioned that a pilot study has been carried [36,38,53,57,67]; however, the findings of the pilot study were not reported.

In regard to validity, many subtypes of validity are available, such as face, content, criterion, and construct validity [144]. In fact, all questionnaire studies should report at least the minimum internal consistency coefficient of Cronbach’s alpha values [21]. For an exploratory or pilot study, it is suggested that reliability should be equal to or above 0.60. In this review, most articles neither report the findings of the pilot study nor of the questionnaire pre-test. Although reliability is important for the study, it is not sufficient unless the validity of the study is provided [21]. In other words, it is crucial for a questionnaire to include a validity index [145]. Thus, the underlying construct of the items in a newly developed questionnaire should be analysed by factor analysis [146] to predict the discriminant and convergent validity. However, only one study was found in this review had carried out the factor analysis [75]. The reliability, content and construct validity of the questionnaire should be carefully examined. It is crucial to harmonise and validate the content of all structured questionnaires, with the aim of reducing the heterogeneity of findings and obtaining quality results based on the questionnaire used for data collection [121,147].

#### Strength and Limitation

This systematic review highlighted the trends of questionnaire-related dengue studies conducted in the past two decades in Malaysia (2000–2020). The substantial number of articles included using data extraction method (over 50 articles) provided an overview of dengue studies in Malaysia. However, this review is not without any limitations. Firstly, it is limited by the inaccessibility of the original questionnaires used, thus restricting a thorough comparison of similarities and differences between different questionnaires. Secondly, the heterogeneity of the findings highlighted in this review might not have resulted from the response per se but due to differences in the studies, such as different modes of statistical analysis, sociodemographic characteristics of the populations under study, scoring systems or cut-off points for the questionnaire, etc. Lastly, the findings in this review should be interpreted with caution due to heterogeneity in the sampling procedure, the number of respondents, questionnaires, scoring method, sample size, and arbitrary cut-offs.

## 5. Conclusions

In conclusion, the systematic review provided some useful insights into recommendations for the control of dengue infection in Malaysia. The efforts of translating knowledge into appropriate attitude and preventive practices regarding dengue should be conducted into more sustainable strategies by the active engagement of communities, inculcation of positive culture, improvements of health campaign (both content materials and personnel), and co-operation with faith-based organisations to spread awareness on dengue prevention and control measures. Next, an early dengue disease recognition and awareness may enhance prompt attendance to medical care in affected populations, thereby reducing mortality and severity of dengue. A good knowledge and attitude towards dengue and vaccination, practices were the most important independent predictors for dengue vaccine acceptance. Ethnicity may also an associated factor to determine dengue vaccine acceptance, but further study is warranted. Besides, it has been discovered that CAM plays an important role in dengue management in Malaysia, especially the rural and remote areas. The integration of traditional herbal medicine in clinical practice may help to complement modern medicine on the basis of sound scientific sources. Thus, efforts are needed to overcome barriers, such as quality control and standardisation issues, pharmacovigilance (e.g., implementation of rules, monitoring, and periodic revision of regulations). Furthermore, behavioural change towards attaining sustainability in dengue preventive practices may be enhanced by fostering comprehensive dengue knowledge and changing health beliefs based on HBM constructs. It is also desirable that different types of communication channels should be applied for reaching out to different types of populations in Malaysia to create awareness on dengue epidemiology. Overall, this review also provides baseline data and reference for designing a reliable questionnaire-based dengue study in the future.

## Figures and Tables

**Table 1:** List of questionnaire-based dengue fever studies in Malaysia (April 2000–April 2020).

Ref	Location of Study	Respondents (N)	Sampling Method and Data Collection Method	Outcome of the Study
[24]	IIUM, Kuantan, Pahang	University students (135)	Convenience sampling and self-administered	Good KAP on dengue amongst respondents.
[25]	Pasir pekan & Kubang kerian, Kelantan	School students (203)	Convenience sampling and self-administered	Respondents from the unflooded area had significantly higher knowledge as compared to the flooded area. Age and dengue history were the primary determinants that influence the high prevention practice.
[26]	Kuala Terengganu, Terengganu	Urban, rural residents and university students (286)	Simple random sampling and face-to-face interview	Dengue awareness programs were moderately effective amongst respondents.
[27]	Bandar Baru Bangi, Selangor	Residential community (152)	Purposive sampling and self-administered	Knowledge of dengue was associated with prevention practice. Television/radio was the predominant source of information about dengue fever. Self-efficacy was significantly associated with house ownership and gender.
[28]	Petaling District, Selangor	Residential community (847)	Convenience sampling and self-administered	High level of awareness on the relationship between climate and dengue. Almost half of the respondents did not know or were not sure how climate can be used to predict dengue.
[29]	CUCMS, Selangor	University student (307)	Convenience sampling and self-administered	KAP scores of respondents increased after health campaigns.
[30]	Perak state, Kedah state, Penang	Residential community (379)	Cluster sampling and face-to-face interview	Majority of respondents showed a favorable interest towards the use of e-learning approach in dengue education.
[31]	Bandar Baru Bangi, Selangor	Residential community (152)	Purposive sampling and self-administered	Significant positive correlation existed between knowledge and practice amongst respondents.
[32]	Selangor & Negeri Sembilan	University students (1144)	Convenience sampling and self-administered	Inadequate knowledge, poor attitude, and poor practice amongst respondents.
[33]	Titiwangsa district, Kuala Lumpur	Residential community (322)	Purposive sampling and face-to-face interview	Perception towards dengue early warning was positive amongst urban residents.∙ Most respondents reported good attitude regarding dengue early warning. Educational level, perception, and attitude are significantly associated with willingness to engage in dengue prevention practice.
[34]	Hulu Langat District, Selangor	Primary school students (171)	Purposive sampling and self-administered	Respondents who had been watching video related to *Aedes mosquitoes*, and the history of dengue infection amongst family members had a significantly better knowledge score.
[35]	Ipoh, Perak	Residential community (259)	Simple random sampling and self-administered	Significant associations between occupation and knowledge, level of education, previous exposure amongst family members and knowledge, and prevention practice.
[36]	Selangor	Residential community (406)	Systematic random sampling and self-administered	People living in non-hotspot areas had better knowledge and attitude than people living in hotspot areas.
[37]	AIMST University, Kedah	Medical university students (200)	Simple random sampling and self-administered	Attitudes was good but practice of dengue control was poor. Significant association between gender and knowledge of dengue serotypes.
[38]	Besut, Terengganu	Residential community (202)	Simple random selection and self-administered	Knowledge and level of education were significantly associated with dengue prevention practice.
[39]	UKMC, Kuala Lumpur	Healthcare provider (330)	Purposive sampling and self-administered	Occupation and level of knowledge were significantly associated with acceptance on Wolbachia as dengue biological control.
[40]	UPM, Serdang, Selangor	Postgraduate international students (327)	Simple random sampling and self-administered	Good knowledge was associated with gender, history of dengue infection and duration of stay and mass media. Good attitude amongst international students was associated with age, mass media and country of origin.
[41]	Penang	Residential community (415)	Convenience sampling and face-to-face interview	Chinese ethnicity, higher dengue knowledge, and vaccination attitude were more likely to accept the vaccine.
[42]	Malaysia	Medical doctor (313)	Universal sampling and self-administered	Long working hours, depression, anxiety, and stress were significantly associated with high degree of burnout syndrome.
[43]	USM, Penang	University students (250)	Purposive sampling and self-administered	Moderate level of KAP with a significant, positive, weak correlation between knowledge, and prevention practice.
[44]	Tok Kenali village, Kelantan	Residential community (132)	Convenience sampling and self-administered	Good level of attitude but poor practice of dengue prevention.
[45]	Sepang, Selangor	Residential community (148)	Universal and simple random sampling and face-to-face interview	Poor knowledge but good prevention practice. Strong correlation of knowledge and attitude.
[46]	UniSZA, Terengganu	Hostel residents (870)	Convenience sampling and self-administered	Age, educational level, knowledge and attitude had significant association with good prevention practice.
[47]	Ipoh, Perak	Residential community (86)	Simple random selection and self-administered	Poor level of awareness and attitude regarding dengue.
[48]	NDUM, Kuala Lumpur	Military cadet & staff (372)	Stratified random sampling and self-administered	High level of knowledge and good attitude but limited practice of dengue prevention.
[49]	Sepang, Selangor	Residential community (305)	Simple random sampling and self-administered	High level of knowledge and good attitude but poor practice of dengue prevention.
[50]	Kuantan, Pahang	Residential community (286)	Simple random sampling and face-to-face interview	No association found between knowledge of dengue and the attitude towards dengue.
[51]	Hospital Taiping, Perak	Inpatients & parents/caretakers (300)	Simple random sampling and self-administered	Significant relationship between knowledge score and socio-demographic factors. Good prevention practice towards dengue was associated with good knowledge.
[52]	Kubang Kerian, Kelantan	Residential community (218)	Simple random sampling and self-administered	Knowledge of dengue signs and symptoms were associated with education level, dengue fever experience and public campaign. Treatment-seeking behaviour were associated with monthly income and history of dengue hospitalisation.
[53]	Malaysia	Medical doctor (864)	Proportionate multistage random sampling and self-administered	Doctors working in private clinic were less likely to utilise the CPG.
[54]	Seremban, Negeri Sembilan	Dengue patients (120)	Purposive random sampling and face-to-face interview	The most frequently used prevention practice for dengue was insecticides aerosol spray, mosquito coils, and mats.
[55]	UPM Serdang, Selangor	International university students (641)	Cluster random sampling and self-administrated	Poor knowledge and negative attitude but good prevention practices.
[56]	Penang General Hospital, Penang	Hospital visitors (337)	Convenience sampling and face-to-face interview	High level of awareness but low levels of dengue prevention practice.
[57]	Terengganu	Residential community (575)	Purposive sampling and face-to-face interview	Age, educational levels, knowledge and attitude were significantly associated with preventive practice against dengue.
[58]	Ampang, Selangor	Residential community (133)	Convenience sampling and self-administered	Good knowledge but poor preventive practice.
[59]	Penang Island, Penang	Residential community (urban, suburban) (202)	Purposive sampling and self-administered	Knowledge of the Aedes biting frequency were associated with dengue infection, lifestyle on the light use, and preventive measures against mosquitoes.
[60]	Malaysia	Malaysian public (2512)	Simple random sampling and phone call interview	Education level, knowledge, and perceived barriers were associated with dengue self-test kit acceptance.
[61]	Kuala Lumpur	Secondary school students (123)	Convenience sampling and self-administered	Significant improvement in the scores after health education.
[62]	UPM Serdang, Selangor	University students (320)	Purposive sampling and self-administered	Good attitude but poor knowledge and prevention practice on dengue.
[63]	Kuala Pilah District, Negeri Sembilan	Residential community (141)	Purposive and simple random selection and face-to-face interview	Risk behaviour of dengue was not covering water container, not turn-over empty water container, not using bed-net at night, not using window mesh or screening, and travelling in and out of locality of epidemic area.
[64]	West Malaysia	Indigenous people (505)	Stratified random sampling and face-to-face interview	Knowledge, perceived barriers to perform dengue prevention, fogging frequency, and perceived susceptibility to dengue were significantly associated with prevention practices.
[65]	Selangor	School children (204)	Convenience sampling and self-administered	Good knowledge and attitudes but poor prevention practices.
[66]	NDUM, Kuala Lumpur	Military cadet (183)	Convenience sampling and self-administered	Good knowledge and attitudes but poor prevention practice.
[67]	Hospital Serdang, Kajang and Kuala Lumpur, Kuala Lumpur & Selangor	Dengue patients (326)	Purposive sampling and face-to-face interview	High prevalence of using CAM, such as isotonic drinks, crab soup, and papaya leaf extract. The most common reason for CAM use was a good impression of CAM from other CAM users.
[68]	Kuala Lumpur	Residential community (1400)	Simple random sampling and telephone-interview	IgG seropositivity in the community was associated with residing in high-rise apartment house, poor perceived severity and susceptibility, and having a neighbour with dengue as a cue to action.
[69]	Gombak, Selangor	Parents or caretakers (866)	Universal sampling and self-administered	Poor ability to recognise fever as life-threatening symptom of dengue in children.
[70]	Jempol, Negeri Sembilan	Residential community (306)	Cluster random sampling and self-administrated	Good knowledge but poor perception towards susceptibility to dengue.
[71]	Rembau & Bukit Pelanduk district, Negeri Sembilan	Residential community (400)	Simple random sampling and face-to-face interview	Knowledge on dengue was associated with age, ethnicity, and educational level.∙ Attitude was associated with ethnicity and educational level. Dengue prevention practice was associated with ethnicity and marital status.
[72]	Selangor	Residential community (322)	Cluster random sampling and face-to-face interview	Level of personal larval control as dengue prevention practices was low. Only those with a good level of attitude towards personal preventive measure and frequent attendance to health campaigns were significantly associated with the good larval control practices.
[73]	UKMC, Banggi, Selangor	Health care providers (61)	Simple random sampling and self-administered	Improvement of all knowledge scores after dengue talks. The improvement was more pronounced in the paramedic group as compared to doctors.
[74]	Selangor & Kuala Lumpur	Residential community (300)	Convenience sampling and self-administered	Attitudes towards dengue were significantly associated with the level of education and employment status. Prevention practice was associated significantly with age, marital status, geographic area, and knowledge.
[75]	Terengganu	Residential community (280)	Simple random sampling and face-to-face interview	Level and strength of self-efficacy mediated knowledge of dengue and dengue preventive behaviours.
[76]	Perak Tengah district, Perak	Residential community (200)	Convenience sampling and self-administered	Age, location and education were statistically significant difference on mean knowledge of dengue.
[77]	Seremban District, Negeri Sembilan	Residential community (321)	Convenience sampling and face-to-face interview	Significant associations were found between knowledge scores of dengue and age, education level, marital status, and occupation.
[78]	Klang Valley, Selangor	Suspected dengue patients (236)	Purposive random sampling and face-to-face interview	Most patients reported of not given any advices on dengue preventive measures by primary care providers.
[79]	Kuala Lumpur	Residential community (133)	Stratified and systematic random sampling and face-to-face interview	Knowledge poor, attitude good, dengue control, and prevention practices poor.
[80]	Kuala Kangsar Perak	Residential community (200)	Simple random sampling and face-to-face interview	Significant association found between knowledge of dengue and attitude towards Aedes control. Mass media is an important source of information regarding dengue fever.

## Data Availability

Data is contained within the article.
